# Does A Medical Consortium Influence Health Outcomes of Hospitalized Cancer Patients? An Integrated Care Model in Shanxi, China

**DOI:** 10.5334/ijic.3588

**Published:** 2018-04-19

**Authors:** Miao Cai, Echu Liu, Hongbing Tao, Zhengmin Qian, Qiang (John) Fu, Xiaojun Lin, Manli Wang, Chang Xu, Ziling Ni

**Affiliations:** 1 Department of Epidemiology and Biostatistics, College for Public Health and Social Justice, Saint Louis University, Saint Louis, Missouri, 63104, US; 2 Department of Health Management and Policy, College for Public Health and Social Justice, Saint Louis University, Saint Louis, Missouri, 63104, US; 3 School of Health Management and Medicine, Tongji Medical College, Huazhong University of Science and Technology, Wuhan, Hubei, 430030, CN

**Keywords:** medical consortium, propensity score matching, the Cox proportional hazard model, cancer patients

## Abstract

**Objective:**

To assess the effect of the medical consortium policy on the outcomes of cancer patients admitted to secondary hospitals in Shanxi, China.

**Method:**

Electronic medical records of lung cancer (n = 8,193), stomach cancer (n = 5,693) and esophagus cancer (n = 2,802) patients hospitalized in secondary hospitals were used. Propensity score matching was used to match each patient enrolled in medical consortium hospitals with a counterpart admitted in non-medical consortium hospitals. Cox proportional hazard models were used to estimate the hazard ratio of patients enrolled different categories of hospitals.

**Results:**

The hazards of lung, stomach and esophageal cancer patients admitted in medical consortium hospitals were consistently and significantly lower than those admitted in non-medical consortium hospitals after adjusting for a number of potential confounders. Lower hazard ratios were associated with lung (hazard ratio (HR) = 0.533, p < 0.001), stomach (HR = 0.494, p < 0.001), and esophagus (HR = 0.505, p < 0.001) cancer patients in medical consortium hospitals.

**Conclusion:**

The medical consortium provides an effective strategy to improve the outcomes of cancer patients in Shanxi, China. The partnerships between top-tier hospitals and grassroots medical services bridge the gap in resources and plays a critical role in the quality of care in China.

## Introduction

With rapid environmental changes, the global disease spectrum has shifted from an infectious disease model to a chronic non-communicable disease model. As for China, the most populous country in the world, cancer has become the leading cause of death and is a major problem in public health [1]. Estimated by Chen et al., approximately 4,292,000 new invasive cancer cases were identified in China in 2015, and an astonishing number of 2,814,000 Chinese people died from cancer in 2015 [1]. Lung cancer, stomach cancer, and esophageal cancer are the most commonly diagnosed cancers among both men and women in China [1].

In view of the high incidence of cancer in this country with a mammoth population size, it is necessary for tertiary hospitals and secondary healthcare institutions to collaborate and provide integrated care for cancer patients. However, the secondary healthcare facilities in China are targeted on common and minor conditions. The capability of these secondary healthcare facilities to cope with cancer patients is limited because of lack of medical expertise, inadequate equipment, and the poor secondary care teamwork [2,3]. The Chinese government has been trying to construct various types of vertical integration among tertiary, secondary, and primary care, wishing that this effort could improve medical expertise and skills of personnel in secondary institutions [4,5,6]. At this point, there are three models of vertical integration: loose integration, medical consortium, and direct management [4].

The medical consortium (Chinese Pinyin: Yi Liao Lian He Ti), established nationwide, has been widely encouraged by the National Health and Family Planning Commission. According to Leut [7], integrated care is defined as the effort to connect the healthcare system, including acute, primary medical and advanced, with other human service systems to improve outcomes. Integrated care generally includes horizontal integration, vertical integration, system integration, organizational integration and others [8]. A medical consortium is a form of vertical integrated care that typically involves one widely recognized tertiary hospital and several secondary hospitals or community health centers and improve the outcomes of patients through the collaboration of different levels of medical care [8,9]. Shared medical professionals and electronic medical records, remote medical treatment and contracted relationships among the medical consortium hospitals make it possible for patients to have continuous care. As described by the general office of the State Council, the primary purpose of establishing medical consortiums is to encourage experienced physicians in tertiary hospitals to work in primary and secondary healthcare facilities, and therefore, instruct medical professionals to improve the quality of care in primary and secondary healthcare facilities [10].

In June 2014, the Health and Family Planning Commission of Shanxi Province initiated the pilot of medical consortium’s construction, including 10 core tertiary hospitals and several secondary hospitals [11]. The first round of medical consortium construction was completed by the end of 2014. Among the 10 core tertiary hospitals that led the first round of medical consortium reform, the only tertiary hospital that was specialized in cancer diagnosis was Shanxi Provincial Cancer Hospital, with 15 secondary hospitals participating in the “cancer medical consortium” and serving approximately 30 million residents in total [12]. As reported by the media, Shanxi Provincial Cancer Hospital tried to help these consortium secondary hospitals in three aspects [12]. Firstly, an expert team was built specifically for this cancer medical consortium. These experts took turns to serve in the consortium secondary hospitals, and contributed to improving staff’s medical skills, consultation of patients, cancer screening, guiding surgery and health education [Shanxi Provincial Hospital]. Secondly, standardized continue education and medical training specialized in cancer treatment were provided to doctors and nurses, aiming at training qualified 1 to 2 doctors and 2 to 4 specialized nurses for each consortium hospital each year. Thirdly, a two-way referral system was established between the leading tertiary hospital and secondary hospitals. Patients with complicated and sever diseases in secondary hospitals were transferred to the tertiary hospital to receive advanced medical care, while those with minor conditions or in the recovery phase in the tertiary hospital were transferred to secondary hospitals to reduce costs.

Although the medical consortium policy has been implemented nationwide in China, very few evidence based on patient level empirical data has been published on the effect of this policy. Therefore, we aim to explore the effects of a medical consortium model on health outcomes of cancer patients in Shanxi, China in this study. Based on the standardized electronic records of lung, stomach, and esophageal cancer patients, we compare the relative risks of patients admitted to secondary hospitals in the medical consortium with those admitted to secondary hospitals not affiliated to medical consortium.

## Methods and Data

### Data

Shanxi province is located in northern China. According to the Statistical Yearbook of Shanxi, there were 36.3 million residents in Shanxi, with 52.6% of them living in urban areas in 2013 [13]. This study is based on the standardized administrative electronic health records (EHRs) in the database of the Health and Family Planning Commission in Shanxi. This EHRs system was standardized and assigned to hospitals all over the country as a compulsory system by the Ministry of Health in China in 2011, with over 200 variables [14]. We collected relevant data involving inpatients over 18-years-old hospitalized in secondary hospitals one year after the medical consortium pilot (from January 2015 to December 2015). The International Classification of Diseases 10th Revision (ICD-10) was used to identify patients diagnosed with lung cancer (C34.000–C34.902), stomach cancer (C16.000–C16.903) and esophageal cancer (C15.000–C15.900). All patients’ and medical practitioners’ personal identifiers (such as name, ID card number, and insurance number) were excluded before the study started. The data contain information about patients’ demographic characteristics (age, gender, marriage status, etc.), diagnosis codes (ICD-10 code for patients’ main diagnosis and up to 10 secondary diagnoses) and outcomes (discharge outcomes during the hospitalization). In total, 8,193 lung cancer patients, 5,693 stomach cancer patients, and 2,802 esophageal cancer patients were identified in the study.

### Propensity score matching

Since patients admitted into the medical consortium hospitals may systematically differ from those in non-medical consortium hospitals in both patient-level and hospital-level characteristics, we used propensity scores to match each patient enrolled in a medical consortium hospital with a similar counterpart in non-medical consortium hospital. Propensity score matching was used to balance and control for observable covariates and reduce the chances of potential selection bias [15]. In essence, propensity score is from a logistic regression, with the binary variable of whether the patient was admitted to a medical consortium hospital being the outcome variable predicted by a number of patient-level and hospital-level covariates. In this study, we constructed the propensity score matching model with five patient-level covariates: gender, age, status of the patient upon admission and whether a surgery was conducted on the patient, C3 index, and five hospital-level covariates: the number of open beds, the number of regular budget physicians, the number of extra contracted physicians, the number of regular budget nurses, and the number of extra contracted nurses.

### Variables

#### Outcome variable

Previous studies based on administrative databases have utilized in-hospital deaths as an outcome since the data following the discharges are normally inaccessible [16,17]. Using this outcome for research could be biased because patients could choose to die at home if the chance of recovery is low. This problem cannot be ignored when studying cancer patients in China. Considering the culture of strong family ties, filial piety, and hospice care, approximately two-thirds of cancer patients in China would prefer to die in their homes [18,19]. Classifying patient’s outcome as a binary of death or non-death would misclassify those who chose to go back home and died shortly. In this study, instead of using a binary variable of death or non-death, we used recovery or non-recovery as the binary part of the outcome variable. The outcome variable in this study includes two parts, a binary variable that indicates the occurrence of the event and a time variable of the time of survival. In the first part, we used a binary variable of recovery at the time of being discharged. We recoded death and not recovered upon discharge as 1 (the event of outcome), the fully recovered and those patients who were better off upon discharge (no event) and unknown discharge status (moving out/dropping off) as 0. In the second part, we used the length of stay (days) in the hospital as the patient’s survival time.

#### Explanatory variables

The explanatory variables in our analysis include 5 patient level covariates, gender, age groups, status upon admission, surgery conducted or not, and C3 index; and 5 hospital level covariates, the number of open beds, the number of regular budget physicians, the number of extra contracted physicians, the number of regular budget nurses, and the number of extra contracted nurses. We recorded the variable age into six categories and defined the 18–44 age group as the reference because this group was thought to be in better physical health and was expected to have better outcomes despite the diagnosis of cancer. Other age groups were classified according to a ten-year interval, and inpatients in higher age groups were expected to have worse outcomes. Gender is also important in predicting outcomes of cancer patients. Significant cancer disparities have been observed between male and female in China [1]. Status upon admission was another factor that could influence the outcomes of patients. Patients classified as “urgent” and “acute” were expected to have worse in-hospital outcomes than those of normal patients. Whether surgery was conducted on a patient or not would influence his or her outcomes due to the risk of complications and nosocomial infections. Comorbidity has an important impact on the outcomes of cancer patients [20]. Several comorbidity indices have been developed for administrative healthcare data to measure the severity of patients’ comorbidities, such as the Charlson Comorbidity Index and the Elixhauser Index [21,22,23,24]. These two indexes have been widely used in predicting patients’ long-term outcomes (one year) and mortality [25,26,27,28]. However, these two indexes are not developed specifically for cancer patients, and the use of the two indexes in the current study was not validated. Thus, we adopted a cancer-specific comorbidity index, C3 (Cancer Care and Comorbidity) index, as the measure of patients’ comorbidity [29]. The C3 index constitutes 42 comorbidity conditions and outperformed the Charlson Index and National Cancer Index for cancer patients [29]. The 42 comorbidities were identified through their corresponding ICD-10 codes [30]. The C3 index is a continuous score ranging between –0.03 and 32.42, where a larger value indicates severer comorbidities.

### Statistical analysis

The statistical analysis was conducted in two steps. Firstly, we used propensity score matching to match each patient enrolled in the medical consortium with a similar counterpart patient hospitalized in a non-medical consortium hospital (one-to-one match). Secondly, we used multivariate Cox proportional hazard models to evaluate the hazard ratios for matched patients enrolled in medical consortiums and those enrolled in non-medical consortium hospitals. The proportional hazards assumption was evaluated by the empirical score process with cumulative sums of martingale-based residuals [31]. We created an interaction term for variables with *p* values less than 5% and the time variable for each model. The *p* values were determined by the Kolmogorov-type supremum test. All data manipulation, statistical analyses, and data visualizations were processed in R studio (Version 1.0.44), while the empirical score process was performed in SAS 9.4.

## Results

Table 1 displays characteristics of lung, stomach, and esophageal cancer patients enrolled in medical consortiums, non-medical consortiums before matching, non-medical consortium hospitals after matching and the percentage of improvement after propensity score matching. As shown in Table 1, the number of patients hospitalized in non-medical consortium hospitals exceeded the number of patients in medical consortium hospitals. Meanwhile, large variations in the characteristics among lung, stomach, and esophageal cancer patients between the medical consortium hospitals and non-medical consortium hospitals before propensity score matching were observed. Patients in non-medical consortiums had a higher C3 index score and there was a lower percentage of normal status patients upon admission than those in medical consortiums, indicating that severe patients were admitted in non-medical consortium hospitals. An average improvement of 57.2% in logistic distance score after propensity score matching was observed, although variations of hospital characteristics were augmented after the matching.

**Table 1:** Descriptive statistics of patient characteristics before and after propensity score matching in different groups.

	Means – Lung cancer				Means – Stomach cancer				Means – Esophageal cancer			

	Treated n = 1,598	unmatched Control n = 6,595	matched Control n = 1,598	percentage of improvement	Treated n = 1,008	unmatched Control n = 4,685	matched Control n = 1,008	percentage of improvement	Treated n = 451	unmatched Control n = 2,351	matched Control n = 451	percentage of improvement

**Gender:**											
**Female**	0.292	0.271	0.299	68.1	0.173	0.202	0.202	–0.8	0.457	0.351	0.486	72.9
**Male**	0.708	0.729	0.701	68.1	0.827	0.798	0.798	–0.8	0.543	0.650	0.514	72.9
**Age group:**											
**18–44**	0.021	0.026	0.027	17.0	0.050	0.035	0.046	72.5	0.007	0.008	0.004	130.0
**45–54**	0.187	0.100	0.109	10.4	0.125	0.106	0.115	48.1	0.106	0.074	0.084	31.6
**55–64**	0.290	0.275	0.304	9.9	0.286	0.273	0.267	–48.2	0.328	0.288	0.277	–28.2
**65–74**	0.348	0.331	0.338	41.4	0.369	0.361	0.387	–114.6	0.344	0.349	0.368	–340.9
**75–84**	0.135	0.230	0.188	43.5	0.152	0.202	0.157	90.1	0.195	0.244	0.237	14.1
**>85**	0.019	0.039	0.034	23.3	0.019	0.024	0.029	–114.3	0.020	0.037	0.029	46.7
**Status upon admission:**											
**Normal**	0.987	0.827	0.977	93.4	0.986	0.834	0.980	96.1	0.998	0.879	0.993	96.2
**Acute**	0.001	0.038	0.004	91.4	0.001	0.032	0.001	100.0	0.000	0.021	0.000	100.0
**urgent**	0.011	0.033	0.019	66.0	0.013	0.029	0.019	62.1	0.002	0.018	0.007	72.4
**other**	0.000	0.102	0.000	100.0	0.000	0.105	0.000	100.0	0.000	0.082	0.000	100.0
**surgery conducted or not**	0.033	0.448	0.031	99.5	0.142	0.494	0.204	82.2	0.087	0.522	0.129	90.3
**C3 index**	0.388	0.620	0.475	62.3	0.315	0.473	0.405	43.2	0.283	0.463	0.421	23.3
**No. of open beds**	427.4	474.0	338.6	–91.0	407.9	429.6	357.5	–131.7	413.4	457.7	354.8	–32.1
**No. of regular budget physicians**	137.9	151.1	117.8	–52.1	122.4	134.5	109.1	–9.5	132.5	137.2	113.4	–307.7
**No. of extra contracted physicians**	137.7	153.2	113.5	–55.6	124.4	132.4	107.1	–116.6	123.9	133.2	105.7	–96.0
**No. of regular budget nurses**	38.3	58.8	26.1	40.7	35.7	51.9	29.8	62.8	36.3	55.6	27.4	54.2
**No. of extra contracted nurses**	154.2	141.2	110.5	–234.4	146.1	128.0	127.6	–2.2	150.9	135.3	120.0	–97.8

**Figure 1:**
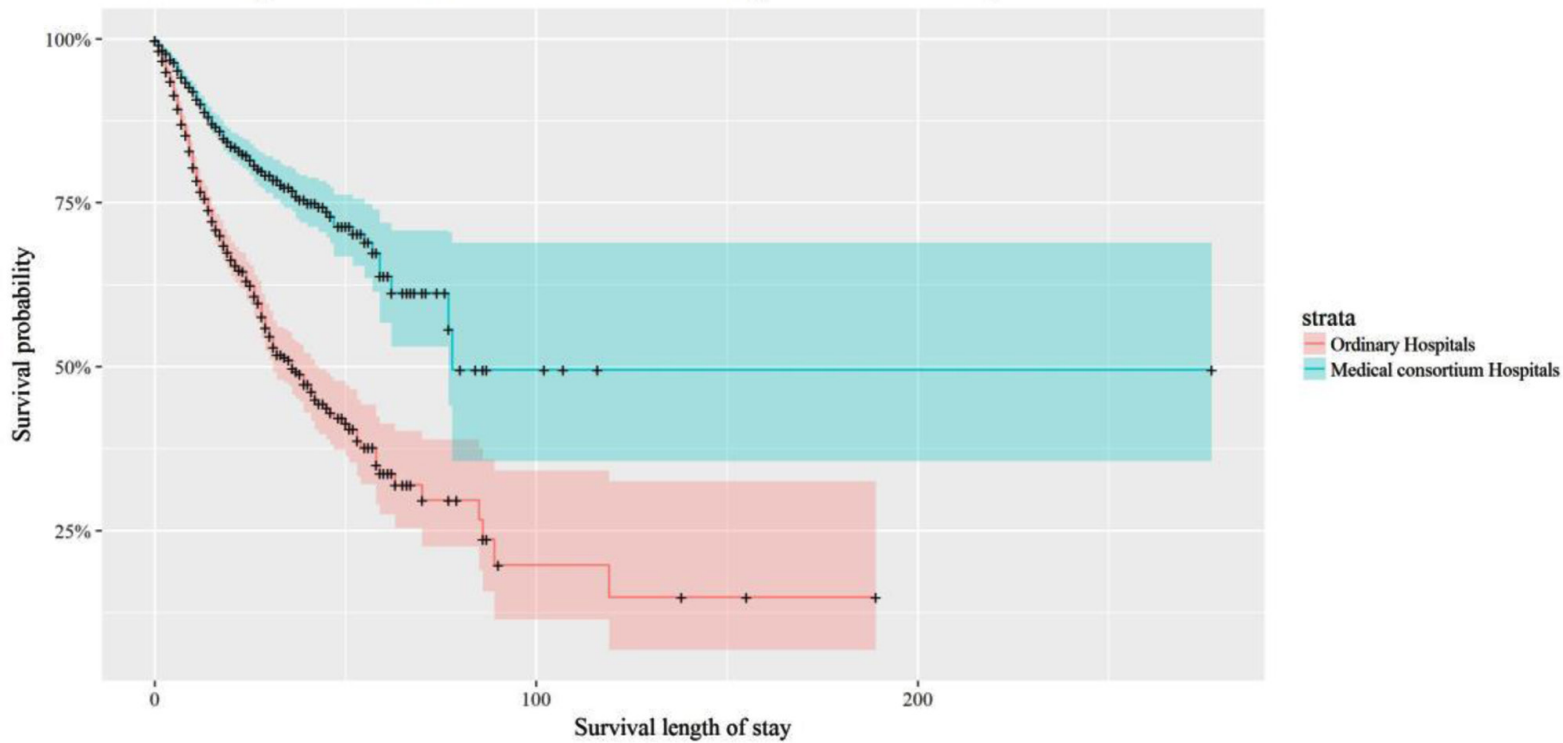
Product-Limit Survival Estimates of Matched Full Sample Patients.

**Figure 2:**
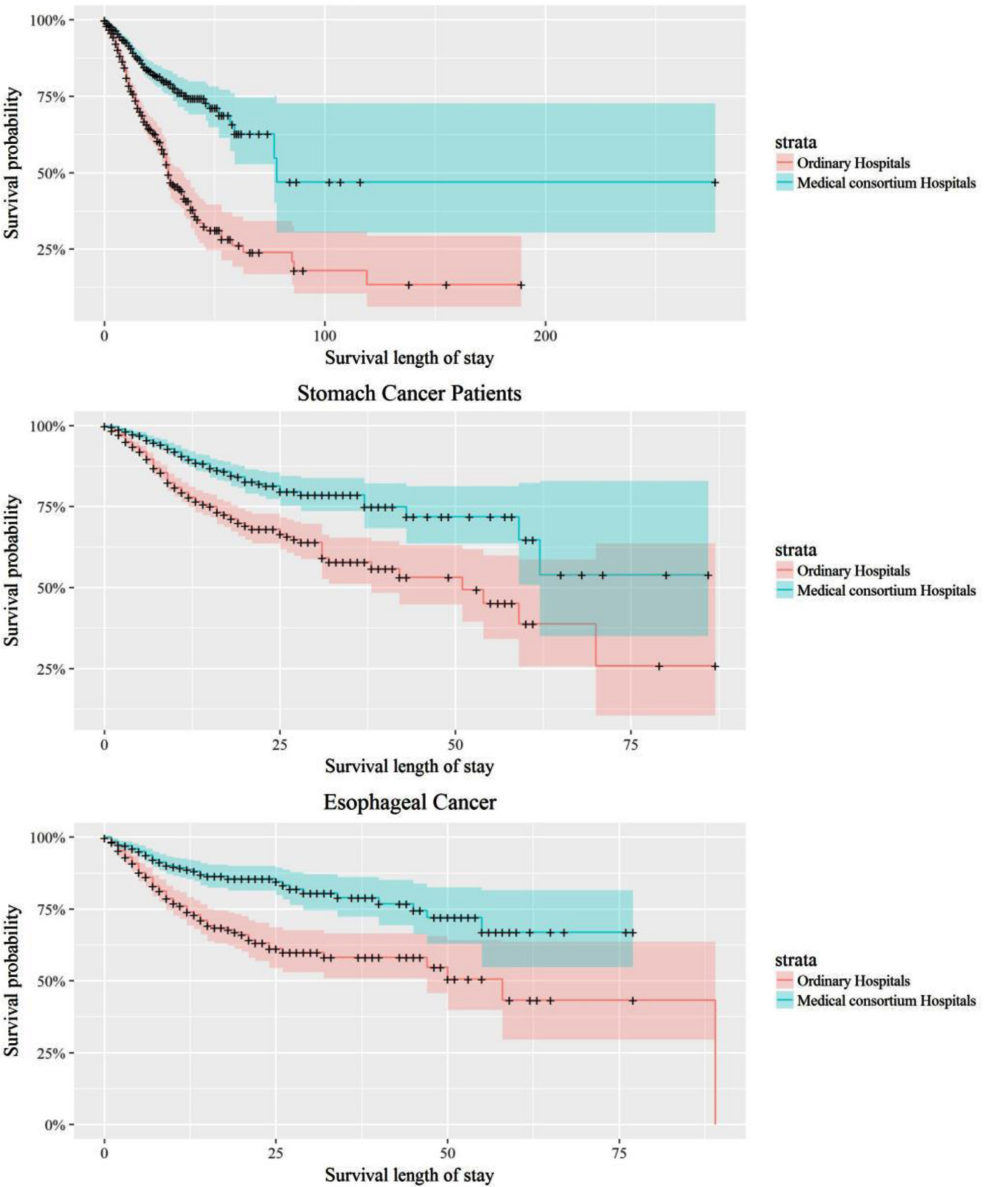
Product-Limit Survival Estimates of 3 Matched Cancer Patients Lung Cancer Patients. *Note*: Strata = 0 denotes patients enrolled in non-Medical Consortium Hospitals, strata = 1 denotes patients enrolled in Medical Consortium Hospitals.

Figure 1 shows the Kaplan-Meier survival curves of matched patients, where the blue lines indicate patients enrolled in medical consortium hospitals, while red lines indicate patients enrolled in non-medical consortium hospitals. The plot indicates that patients enrolled in medical consortiums consistently had higher survival probabilities, compared with those in non-medical consortium hospitals at the same survival time, regardless of types of cancers. Similarly, Figure 2 shows the Kaplan-Meier survival curves of the lung, stomach, and esophageal cancer patients with matched data. The plot indicated that patients enrolled in medical consortiums consistently had higher survival probabilities, compared with those in non-medical consortium hospitals at the same survival time across three types of cancers. Nonetheless, the confidence intervals had small intersections after 50 days.

Table 2 illustrates the estimates of the Cox hazard models for lung, stomach, and esophageal cancer matched patients. After checking the proportional hazard assumptions with the empirical score process, it was found that the C3 variables for both lung cancer patients and stomach cancer patients did not meet the proportional hazard assumption. Therefore, an interaction term was added at the end of the variable column for the lung cancer and stomach cancer groups. Lower hazard ratios were associated with patients enrolled in medical consortium hospitals across lung cancer (hazard ratio = 0.533, p < 0.001), stomach cancer (hazard ratio = 0.494, p < 0.001) and esophageal cancer patients (hazard ratio = 0.505, p < 0.001).

**Table 2:** Maximum likelihood estimates of multivariable Cox hazard model.

Parameter	Lung cancer				Stomach cancer				Esophageal cancer			

	Hazard Ratio	Pr > ChiSq	95% Hazard Ratio Confidence Limits		Hazard Ratio	Pr > ChiSq	95% Hazard Ratio Confidence Limits		Hazard Ratio	Pr > ChiSq	95% Hazard Ratio Confidence Limits	

**Non-medical consortium hospitals**	Ref.	Ref.	Ref.	Ref.	Ref.	Ref.	Ref.	Ref.	Ref.	Ref.	Ref.	Ref.
**Medical consortium hospitals**	0.533	<.001	0.439	0.648	0.494	<.001	0.386	0.634	0.505	<.001	0.361	0.708
**Age groups:**											
**18–44**	Ref.	Ref.	Ref.	Ref.	Ref.	Ref.	Ref.	Ref.	Ref.	Ref.	Ref.	Ref.
**45–54**	1.122	0.693	0.633	1.990	0.847	0.595	0.460	1.560	0.244	0.065	0.055	1.089
**55–64**	1.075	0.792	0.627	1.843	0.697	0.210	0.396	1.226	0.168	0.017	0.039	0.727
**65–74**	1.132	0.650	0.662	1.934	0.801	0.427	0.463	1.385	0.214	0.039	0.049	0.924
**75–84**	1.329	0.311	0.767	2.304	0.680	0.202	0.376	1.229	0.257	0.071	0.059	1.125
**>85**	1.224	0.542	0.640	2.341	0.913	0.815	0.424	1.964	0.513	0.428	0.098	2.673
**Gender**											
**Female**	Ref.	Ref.	Ref.	Ref.	Ref.	Ref.	Ref.	Ref.	Ref.	Ref.	Ref.	Ref.
**Male**	0.895	0.247	0.742	1.080	0.907	0.493	0.685	1.200	1.134	0.439	0.825	1.557
**Status upon admission**											
**Ordinary**	Ref.	Ref.	Ref.	Ref.	Ref.	Ref.	Ref.	Ref.	–	–	–	–
**Acute**	5.657	<.001	2.492	12.841	7.902	0.041	1.086	57.506	–	–	–	–
**Urgent**	2.696	<.0001	1.750	4.155	2.447	0.010	1.236	4.843	–	–	–	–
**Surgery conducted or not**	0.543	0.023	0.320	0.921	0.596	0.006	0.411	0.864	1.307	0.334	0.760	2.248
**C3 Index**	0.994	0.935	0.856	1.153	0.981	0.875	0.769	1.251	0.970	0.818	0.750	1.255
**No. of open beds**	0.998	<.001	0.997	0.999	0.998	0.002	0.996	0.999	0.999	0.209	0.997	1.001
**No. of regular budget physicians**	1.006	<.001	1.003	1.008	1.009	0.000	1.004	1.014	1.004	0.196	0.998	1.009
**No. of extra contracted physicians**	1.013	<.001	1.008	1.018	1.009	0.065	0.999	1.018	0.998	0.765	0.986	1.010
**No. of regular budget nurses**	0.995	<.001	0.993	0.998	0.989	<.001	0.984	0.993	0.997	0.242	0.991	1.002
**No. of extra contracted nurses**	0.993	<.001	0.991	0.995	0.999	0.531	0.995	1.002	0.997	0.192	0.993	1.001
**Interaction**	1.010	0.002	1.004	1.016	1.019	0.014	1.004	1.035	–	–	–	–

Ref. denotes the reference group; The interaction term is the product of the variable that does not meet the proportional hazard assumption and the survival time variable.

## Discussion

The medical consortium policy has not been widely explored and promoted in China until five years ago [32]. The standardized electronic medical record system in Shanxi province makes it possible for us to evaluate the effect of the medical consortium on cancer patients’ health outcomes. To our knowledge, the current study is the first attempt to explore the effects of a medical consortium policy on patients using quantitative data in China. In this study, we found that the hazards of getting unfavorable outcomes for lung, stomach and esophageal cancer patients admitted in medical consortium hospitals were consistently and significantly lower than those admitted in non-medical consortium hospitals, after adjusting for a number of potential patient-level and hospital-level confounders.

According to the official document released by the Health and Family Planning Commission in Shanxi, the medical consortium pilot in 2014 focused on eight key fields: key clinical specialties, pair-up support of urban hospitals on rural hospitals, multisite practice of physicians, two-way referrals, centralized medical examination, telemedicine and innovation in the medical payment system [9]. The effective implementation of these aspects by the leading hospitals was crucial to the positive effect on patients. This implementation is especially important for patients diagnosed with cancer since cancer is a complicated chronic disease that routinely requires medical expertise and multidisciplinary coordination [16,33,34]. The expertise and experience of specialists from the leading hospitals in the medical consortium could provide valuable lessons for physicians in secondary hospitals [35]. Meanwhile, patients could have access to advanced medical equipment and therapies in the leading hospitals by virtue of telemedicine or two-way referrals. These ways are all positive aspects and potential reasons for the success of a medical consortium.

As mentioned in the introduction section, the leading hospital, Shanxi Provincial Cancer Hospital, has been taking three actions to improve the medical quality and service in secondary consortium hospital. As from our understanding, the expert team built specifically for this cancer medical consortium could be the primary reason for the significant improvement in the outcomes of cancer patients in these consortium secondary hospitals. According to the statistics by Shanxi Provincial Hospital, the Shanxi Provincial Hospital has provided specialty consulting service for 320 cases, and guided 30 surgeries on the spot in consortium secondary hospitals by the end of March in 2015 [12]. The collaboration between these experts from Shanxi Provincial Hospital and physicians in consortium secondary hospitals given local people the access to quality tertiary service without travelling all the way to the metropolitans, and could possibly justify most part of the positive results in this study. Further education and specialized training could have long-term effects on the medical workers in consortium secondary hospitals, however, we could suspect that it will not have such significant improvement on patients’ outcome within just one year after the pilot. The two-way referral system is to achieve the hierarchical tertiary care system, but the possibility that patients in consortium secondary hospitals are systematically less severe than patients in non-medical consortium hospitals has been ruled out by the propensity score matching in the first stage of analysis.

Despite the favorable effects for medical consortium on the outcomes of cancer patients found in this study, the medical consortium is far from a panacea for cancer patients. Driven by the popular medical consortium policy implemented nationwide and under the administrative pressure, leading hospitals send their best experts and lend their most advanced medical equipment to county and secondary hospitals without sufficient reimbursements. The experts and advanced equipment are underused in various regions, typically with less population density and purchase power. This situation could have contributed to higher economic values for the leading hospitals. Administrative pressure cannot motivate them to play the active role in long-term. One plausible explanation may be the two-way referral mechanism by which the leading hospitals could obtain more patients through the alliances with county and secondary hospitals. These alliances are exactly why we should remain cautious about the medical consortium policy. When the only incentive for leading hospitals in the medical consortium group is obtaining more patients from their alliances, the leading hospitals are essentially expanding their territories by taking advantage of this policy which compromises the competition in the hospital market. On the other hand, evidence from the United States, England, Netherland, and China indicated that the competition could improve medical quality and health outcomes [36,37,38,39]. If the major incentive for the leading hospital is expanding their sources of patients, it is unlikely that a medical consortium policy can generate positive effects on patients’ outcomes in the long run.

This study has four limitations. A standardized hospital information system was not established on a large scale before the implementation of the medical consortium policy. We were unable to collect data from 2013 and 2014. Thus, we could not examine the causal effect of the medical consortium policy on patients’ outcomes. Secondly, patients’ data following discharges were inaccessible to the current study, which may lead to potential biases. Thirdly, the stages of the cancer cannot be identified because we are using a general electronic health record database, not a database specifically for cancer patients. Lastly, there is possibility that the patients might be transferred from the tertiary hospital to secondary hospitals in that medical consortium. We could not identify these transferred patients in this study because individual patient identifier has been deleted before we have access to the data.

## Conclusion

Implementing the medical consortium policy in Shanxi has led to positive effects on cancer patients’ health outcomes. Policymakers should learn from the experience of establishing cancer medical consortiums in Shanxi, China and pilot a medical consortium model for patients diagnosed with other diseases and in other regions in China.
